# Impact of clinical pharmacist-led intervention for drug-related problems in neonatal intensive care unit a randomized controlled trial

**DOI:** 10.3389/fphar.2023.1242779

**Published:** 2023-08-14

**Authors:** Nadir Yalçın, Merve Kaşıkcı, Hasan Tolga Çelik, Karel Allegaert, Kutay Demirkan, Şule Yiğit

**Affiliations:** ^1^ Department of Clinical Pharmacy, Faculty of Pharmacy, Hacettepe University, Ankara, Türkiye; ^2^ Department of Biostatistics, Faculty of Medicine, Hacettepe University, Ankara, Türkiye; ^3^ Division of Neonatology, Department of Child Health and Diseases, Faculty of Medicine, Hacettepe University, Ankara, Türkiye; ^4^ Department of Pharmaceutical and Pharmacological Sciences, Leuven, Belgium; ^5^ Department of Development and Regeneration, Leuven, Belgium; ^6^ Department of Hospital Pharmacy, Erasmus Medical Center, Rotterdam, Netherlands

**Keywords:** clinical pharmacist (CP), drug-related problems (DRPs), medication errors, adverse drug reactions, drug-drug interactions (DDIs), pharmaceutical care, pharmacotherapy optimization

## Abstract

**Introduction:** Drug-related problems (DRPs) incidence is higher in neonatal intensive care units (NICUs), compared to other pediatric wards due to aspects like off-label medications, pharmacokinetic/dynamic variability, or organ dysfunction/immaturity. This study aimed to determine whether and to what extent a clinical pharmacist intervention improves medication safety and prevents DRPs [medication errors (MEs), adverse drug reactions (ADRs), drug-drug interactions (DDIs)].

**Methods:** A prospective, randomized, double blind, controlled study in NICU-admitted neonates was conducted. NICU patients were randomly assigned to the intervention (clinical pharmacist-led) (IG) or control group (standard care such as clinical diagnosis, pharmacotherapy) (CG). The clinical pharmacist was involved in the IG to identify-prevent-intervene MEs, or identify and monitor ADRs and DDIs. The primary outcome was the number of neonates who developed at least one DRP compared with those seen across IG and CG. Secondary outcomes included length of hospital stay, total number of drugs or DRP type.

**Results:** Neonates were randomly assigned to CG (n = 52) or IG (n = 48). In total, 45%, 42%, and 16% of patients had at least 1 MEs, ADRs, and clinically significant DDIs, respectively. The number of patients with at least 1 ME was 28 (53%) and 17 (35%) in the CG and IG (*p*>0.05). The median (range) number of ME was higher in CG [1 (0–7)] than in IG [0 (0–4)] (*p* = 0.003). Applying regression analysis, the CG had 2.849 times more MEs than the IG (*p*<0.001). Furthermore, the number of patients (CG to IG) with at least one detected ADR or clinical DDI was 19 (36%) to 23 (47%) (*p*>0.05) and 4 (7%) to 12 (25%), respectively (*p* = 0.028).

**Conclusion:** Clinical pharmacist availability to systematically and standardized identify, prevent and resolve DRPs among NICU patients is effective. Daily detailed clinical pharmacist observations and interventions enables prevention and monitoring of DRPs.

**Clinical Trial Registration** ClinicalTrials.gov, identifier NCT04899960.

## Introduction

Neonates are highly vulnerable to drug-related problems (DRPs) [medication errors (MEs), adverse drug reactions (ADRs), and drug-drug interactions (DDIs)]. This is because of their extensive exposure to medications in the neonatal intensive care unit (NICU), the lack of evidence on personalized interventions in neonates and the paucity of neonate-specific formulations (Chedoe et al., 2007; Shaniv et al., 2023). This complexity proliferates in NICUs, with frequent usage of off-label and unlicensed medications, pharmacokinetic/pharmacodynamic variability, organ dysfunction/immaturity, genetic variability, and the need to manipulate available formulations.

In a prospective longitudinal observational study, DRPs were identified in most neonates (60.5%) admitted in a NICU (Leopoldino et al., 2019). A study from the United Kingdom reported that the DRPs incidence was higher in NICUs (25.7%), compared to pediatric intensive care units (25.0%) or other pediatric wards (18.7%) (Rashed et al., 2014). Another study found that 45.2% of pediatric patients experienced DRPs, 80.3% of which were assessed as preventable (Rashed et al., 2012). In a systematic review on the prevalence of MEs and preventable adverse drug events (ADE), a range from 4 to 35.1 and 0.47 to 14.38 per 1,000 patient-days in NICUs was reported for ME and ADE respectively (Alghamdi et al., 2019).

Clinical pharmacists are healthcare professionals with specific expertise, instrumental to a multidisciplinary team effort required to optimize pharmacotherapy. This includes adjustments in dose, interval, administration time, infusion rate, preparation, storage, compatibility, monitoring, simplification of the regimen, or finding alternative medications. Clinical pharmacists are also involved in identification and prevention of ADRs, and assessment and mitigation of potential/clinically significant drug-drug interactions (pDDIs/cDDIs) in NICUs (Kara et al., 2021).

Although there are many reports on how and to what extent clinical pharmacists ensured drug safety and reported DRPs in the NICU since the 1980s, we are not aware of a randomized clinical trial (RCT) exploring and quantifying the effect of a clinical pharmacist intervention in the NICU setting (Campino et al., 2008; Palmero et al., 2019; King et al., 2023). Therefore, the objective of this RCT was to determine whether a clinical pharmacist intervention improves medication safety and prevents DRPs (MEs, ADRs, and DDIs) in neonates admitted to the NICU.

## Methods

### Study setting

This prospective, double blind, randomized study was conducted at a tertiary care Children’s Hospital NICU with a 22-bed capacity in Turkey between November 2022 and January 2023.

The double-blind construct hereby refers to the fact that neither parents/legal guardians, nor NICU physicians or nurses of the multidisciplinary team were aware of group allocation, nor the type of intervention and aim of the study.

In patients in the intervention group (IG), interventions were suggested by a clinical pharmacist to the physicians or nurses. IG neonates were evaluated by a clinical pharmacist who evaluated the patients’ therapies to detect, prevent and manage DRPs (i.e., MEs, ADRs, and DDIs). In contrast, the control group (CG) were cared for by the routine hospital pharmacy services in terms of medication order control (standard practices) and a neonatologist in terms of detecting and monitoring of ADRs and DDIs, and did not include a dedicated clinical pharmacist. These routine clinical pharmacy services were already provided for 2 years prior to initiation of the study.

### Drug-related problems, checklists and interventions

DRPs were prospectively, simultaneously, and daily reviewed during hospitalization by the clinical pharmacist (IG) and neonatologist (CG) respectively, using the same, standardized checklists and tools to ensure standardization (Supplementary Table S3–S9). A blinded independent neonatologist (senior consultant) was involved in clinical diagnosis, indicating pharmacotherapy, drug selection, dosage adjustment, monitoring and approving all recommendations for each neonate in both groups. The checklists were based on current literature and databases and agreed by the authors prior to the study, and covered clinical assessment, causality and severity assessment of ADRs, and severity assessment of cDDI.

Changes in clinical assessments (physical examination, vital signs or laboratory data compared to the baseline) and current literature were taken into account to determine whether ADRs and DDIs were actually drug-related (causality). The Du’s tool and drug interaction probability scale were hereby applied (Horn et al., 2007; Du et al., 2013) (Supplementary Tables S4, S7). ADRs severity was determined by neonatal adverse event severity scale designed by the International Neonatal Consortium (Salaets et al., 2019) (Supplementary Table S5). Furthermore, cDDIs severity was determined using the *UpToDate* (*Lexicomp* ^*®*^) drug interaction database (Supplementary Table S8) (Truven Health, 2023).

The severity of MEs was defined according to the US National Coordinating Council for Medication Error Reporting and Prevention (NCC MERP) as “any preventable event that may cause or lead to inappropriate medication use or patient harm while the medication is under the control of the healthcare professional, patient, or consumer. The authors had the choice between the NCC MERP index categories B (no harm, an error occurred but the error did not reach the patient; C (no harm, an error occurred that reached the patient but did not cause patient harm), and D (no harm, an error occurred that reached the patient and required monitoring to confirm that it resulted in no harm to the patient and/or required intervention to preclude harm) in line with clinical assessment (Hartwig et al., 1991).

In the IG, the clinical pharmacist performed proactive interventions in the NICU to identify DRPs by providing recommendations to physicians (prescriptions, monitoring) or nurses (administration, preparation). To do so, a specific and standardized checklist was used, with focus on *prescription* (inappropriate drug, unit, dose, dose interval, infusion rate, diluent), *preparation* (inappropriate drug, occupational safety, and storage), *administration* (omission, extra dose, inappropriate time, infusion, technique) and *monitoring* (physical, vital, laboratory, therapeutic drug monitoring). This checklist was used to prospectively collect routine daily follow-up in IG by the clinical pharmacist and in CG by the neonatologist (Supplementary Table S3–S9). *Micromedex* ^*®*^ *Neofax* and *UpToDate* (*Lexicomp* ^*®*^) were hereby used as reference databases on drug information. For quantitative parameters (like dose, time, infusion rate), a margin of error more than 5% was applied to be qualified as MEs.

**FIGURE 1:**
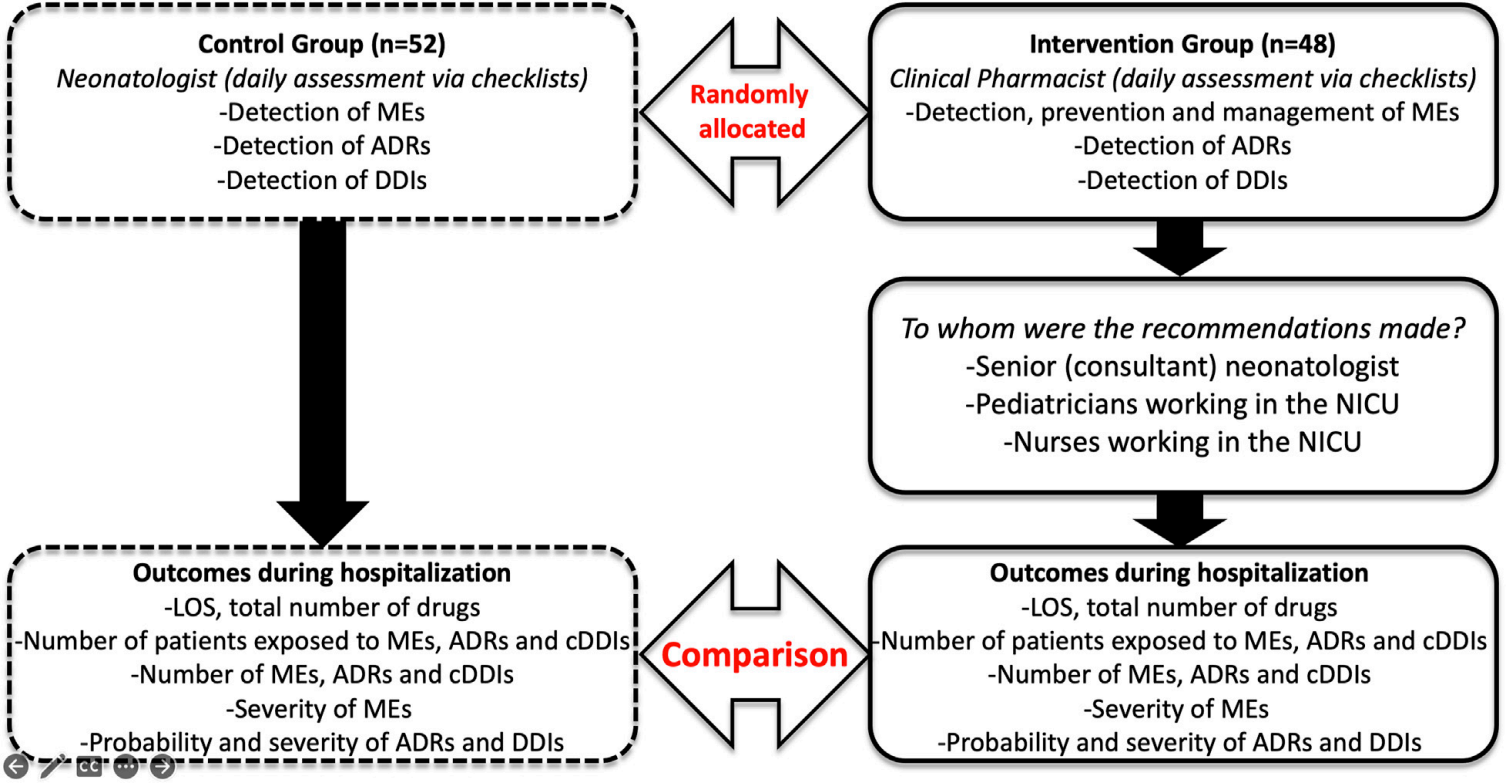
Flow chart of the study procedure.

Considering the detected MEs in the check lists obtained, appropriate recommendations were provided to the physicians or nurses in the IG. In the IG, all recommendations aimed to be approved by the physicians and nurses to be implemented. Followed by these appropriate recommendations were provided, it was checked daily whether the interventions were implemented out during the prescribing, preparation, administration, or monitoring process. Data in non-survivors during the hospitalization period were not included in analysis. The steps for randomization and assessment of outcomes summarized in the flow chart (Figure 1).

### Ethics, study participants, and randomization

The study was conducted according to the guidelines of the Declaration of Helsinki, and approved by the Ethics Committee of Hacettepe University. Before enrolment, informed consent was obtained from the parents/legal guardians.

All patients admitted the NICU of a tertiary care children’s hospital during study period were screened. Patients aged between 0–28 days who started on at least one systemic medication within the first 24 h after admission to the NICU were considered. Demographical, clinical, and drug administration data were obtained from routine daily follow-up for each neonate. International Classification of Diseases 10th Revision (ICD-10) codes for diagnoses, Anatomical Therapeutic Chemical (ATC) codes for categorization of prescribed medications were used. Each eligible neonate was randomly assigned at baseline to either the IG or CG group (1:1) by simple randomization using R program (version 3.6.3, http://www.rproject.org) with sample function. In terms of reproducibility for the random numbers, the seed number was set at 1,234 in this program.

### Assessment of outcomes

The primary outcome was the number of neonates who developed at least one DRP (MEs, ADRs, pDDIs, and cDDIs) during neonatal stay when compared with those seen across the IG and CG. For that, checklists and follow-up forms developed in agreement with the neonatologist and clinical pharmacist and specific probability and severity tools from the current literature were used in both groups. For DDIs, *UpToDate* (*Lexicomp* ^*®*^) database was used to identify pDDIs. For monitoring of cDDIs, follow-up forms, clinical, and laboratory findings were used during the exposure every day for the duration of exposure to the pDDI in the light of UpToDate database information. Secondary outcomes as covariates included mean length of hospital stay (LOS), total number of drugs, total number of each DRP type. To assess the detected DRPs, the clinical pharmacist took responsibility to assess the DRPs in all neonates of both groups using a definition and classification of DRPs following a modified version of the Hepler-Strand classification system (Hepler and Strand, 1990) (Supplementary Table S1).

We also collected infant acuity scores (Supplementary Table S2) to determine the difference in the level of care between both groups at admission (Tubbs-Cooley et al., 2019). Each time an infant was admitted to the NICU, these standard illness scores were calculated to determine the level of nursing care needed within 24 h of admission. The score includes clinical indicators of the level of nursing care provided, such as mode of ventilation, frequency and mode of feeding, quantity and type of infusions, and procedures performed. Higher scores indicate more intensive nursing care; the range for each indicator varies depending on the number and type of items evaluated (1–5 levels) (Tubbs-Cooley et al., 2019).

### Statistical analysis

It was planned to include 100 neonates admitted to the NICU within the stipulated timeframe, based on an effect size of 0.50 (for differences between two-independent means), a power of 80%, and a margin of error of 5% (G* Power 3.1 Statistical Power Analysis). After data extraction, continuous variables were defined as the mean (standard deviation, SD) and median (range). Categorical variables were defined as the frequency and percentage. The normality of continuous variables was tested using the Shapiro–Wilk test. The relations between categorical variables were evaluated with χ2 test. When parametrical test assumptions were met, comparisons between two independent groups were performed with independent sample *t*-test. Otherwise, Mann-Whitney U test was used. Lastly, Poisson regression analysis was run to compare both groups in terms of all DRPs prediction. For all tests, *p* < 0.05 was considered statistically significant. All analyses were carried out in the *IBM SPSS Statistics Version 23* software.

## Results

During the study period (November 2022—January 2023), 109 neonates were screened, 100 neonates fulfilled the inclusion criteria and were recruited for the study, covering 2,780 patient days and 995 medication orders. Nine neonates were excluded because they did not survive (n = 4, 3.6%) or did not receive systemic medication (n = 5, 4.6%) after enrolment. One hundred patients were randomly allocated to either the CG (n = 52) or IG (n = 48).

### Clinical characteristics

There were no significant differences between both groups in terms of patient and maternal demographics, including the acuity level (Table 1). Half of the patients were male, 64% were preterm birth (<37 weeks gestational age) and 56% of the patients had a low birth weight (<2,500 g).

**TABLE 1:** Clinical characteristics in both groups.

Variables	Control group (n = 52)	Intervention group (n = 48)	*p* value
Gender, female, n (%)	28 (53)	22 (45)	>0.05
Gestational age, n (%)			
Very preterm (28 to 32 weeks)	11 (21)	11 (22)	>0.05
Moderate to late preterm (32 to 37 weeks)	23 (44)	19 (39)	
Term (≥37 weeks)	18 (34)	18 (37)	
Small for gestational age, n (%)	17 (32)	13 (27)	>0.05
Birth weight, n (%)			
Extremely low birth weight (<1,000 g)	4 (7)	2 (4)	>0.05
Very low birth weight (1,000 to 1,500 g)	6 (11)	5 (10)	
Low birth weight (1,500 to 2,500 g)	21 (40)	18 (37)	
Normal birth weight (>2,500 g)	21 (40)	23 (47)	
Maternal age (year), median (range)	31 (18–44)	30 (19–43)	>0.05
Multiple birth, n (%)	9 (17)	5 (10)	>0.05
Cesarean Section, n (%)	48 (92)	42 (87)	>0.05
Clinical diseases, n (%)			
Complications of labor and delivery	30 (57)	22 (45)	>0.05
Diseases of the circulatory system	9 (17)	13 (27)	
Diseases of the digestive system	3 (5)	4 (8)	
Others	10 (19)	9 (18)	
Type of respiratory support, n (%)			
Invasive mechanical ventilation	16 (30)	12 (25)	>0.05
Non-invasive mechanical ventilation	13 (25)	19 (39)	
None	23 (44)	17 (35)	
Surgery, n (%)	17 (32)	19 (39)	>0.05
Parenteral nutrition treatment, n (%)	32 (61)	31 (64)	>0.05
Infant Acuity Level, n (%)			
Requiring intermediate care	7 (13)	7 (14)	>0.05
Requiring intensive care	27 (51)	26 (54)	
Requiring multi-system support	11 (21)	7 (14)	
Unstable, requiring complex critical care	7 (13)	8 (16)	
Length of stay (day), median (range)	11.5 (3–112)	17 (3–134)	>0.05

### Prescribed medications

The most commonly prescribed drugs in terms of the number of patients in the CG and IG were alimentary tract and metabolism (94% vs. 100%), anti-infectives for systemic use (65% vs. 77%), and nervous system drugs (38% vs. 47%), respectively. However, there was no significant difference between both groups in terms of number of patients for all medications used and median number of total prescribed drugs (Table 2). The drugs included in these pharmacological groups according to each ATC code during the study period are provided in Supplementary Table S10.

**TABLE 2:** Comparison of prescribed medications (by Anatomical Therapeutic Chemical code) during hospitalization in both groups.

	Control group (n = 52)				Intervention group (n = 48)			
Medications	Rx	n	Row %	Column %	n	Row %	Column %	*p* value
Anti-infectives for systemic use	X	18	62	34	11	37	22	>0.05
	✓	34	47	65	37	52	77	
Systemic hormonal preparations	X	42	53	80	37	46	77	>0.05
	✓	10	47	19	11	52	22	
Nervous system	X	32	56	61	25	43	52	>0.05
	✓	20	46	38	23	53	47	
Blood and blood-forming organs	X	45	55	86	36	44	75	>0.05
	✓	7	36	13	12	63	25	
Alimentary tract and metabolism	X	3	100	5	-	-	-	>0.05
	✓	49	50	94	48	49	100	
Cardiovascular system	X	36	56	69	28	43	58	>0.05
	✓	16	44	30	20	55	41	
Respiratory system	X	35	55	67	28	44	58	>0.05
	✓	17	45	32	20	54	41	
Sensory organs	X	48	52	92	43	47	89	>0.05
	✓	4	44	7	5	55	10	
Total, median (range)		5 (1–32)			9.5 (1–34)			>0.05

Rx prescription of relevant medications, ✓ prescribed, X not prescribed, Row distribution of prescribed medication between groups, Column distribution of prescribed medication within groups.

### Assessment of outcomes

#### Medication errors

The number of patients with at least 1 ME was 28 (53%) in the CG, and 17 (35%) in the IG. MEs were more commonly detected in the CGl than in the IG (*p* = 0.003). The most common physician-related ME was dosing during prescription (9%), and the most common nurse-related ME was related to the time of administration (41%). In the IG, the correct drug (*p* = 0.027) and dosage (*p* = 0.032) at prescription, the correct method of preparation (*p* = 0.027) and the correct duration of infusion (*p* = 0.013) at administration were more accurate (Table 3). Furthermore, 40 clinical pharmacist-led recommendations were provided to the physicians and nurses in the IG for DRPs that emerged in the logistics, preparation, dose, administration technique, and monitoring process. All of them were accepted by physicians and nurses (Supplementary Table S11. According to the NCC MERP criteria, we did not observe any serious ME in any of the patients in the study. Also, there were no significant differences in serious ME between groups.

**TABLE 3:** Comparison of medication errors during hospitalization in both groups.

	Type of ME	Number of patients, n (%)		
Physicians	Prescription	Control group (n = 52)	Intervention group (n = 48)	*p* value
	Inappropriate drug	6 (11)	-	0.027
	Inappropriate unit	-	-	-
	Inappropriate dose	8 (15)	1 (2)	0.032
	Inappropriate dose interval	3 (5)	1 (2)	>0.05
	Inappropriate infusion rate	5 (9)	-	>0.05
	Inappropriate diluent	1 (1)	-	>0.05
	Monitoring			
	Physical	-	-	-
	Vital	-	-	-
	Laboratory	2 (3)	-	>0.05
	TDM	5 (9)	-	>0.05
Nurses	Preparation			
	Inappropriate drug	6 (11)	-	0.027
	Inappropriate occupational safety	2 (3)	-	>0.05
	Inappropriate storage	1 (1)	-	>0.05
	Administration			
	Dose omission	-	-	-
	Extra dose	-	-	-
	Inappropriate time	20 (38)	21 (43)	>0.05
	Inappropriate infusion	7 (13)	-	0.013
	Inappropriate technique	7 (13)	2 (4)	0.163
Presence of at least 1 ME		28 (53)	17 (35)	>0.05
Number of ME type, median (range)		1 (0–7)	0 (0–4)	0.003

**FIGURE 2:**
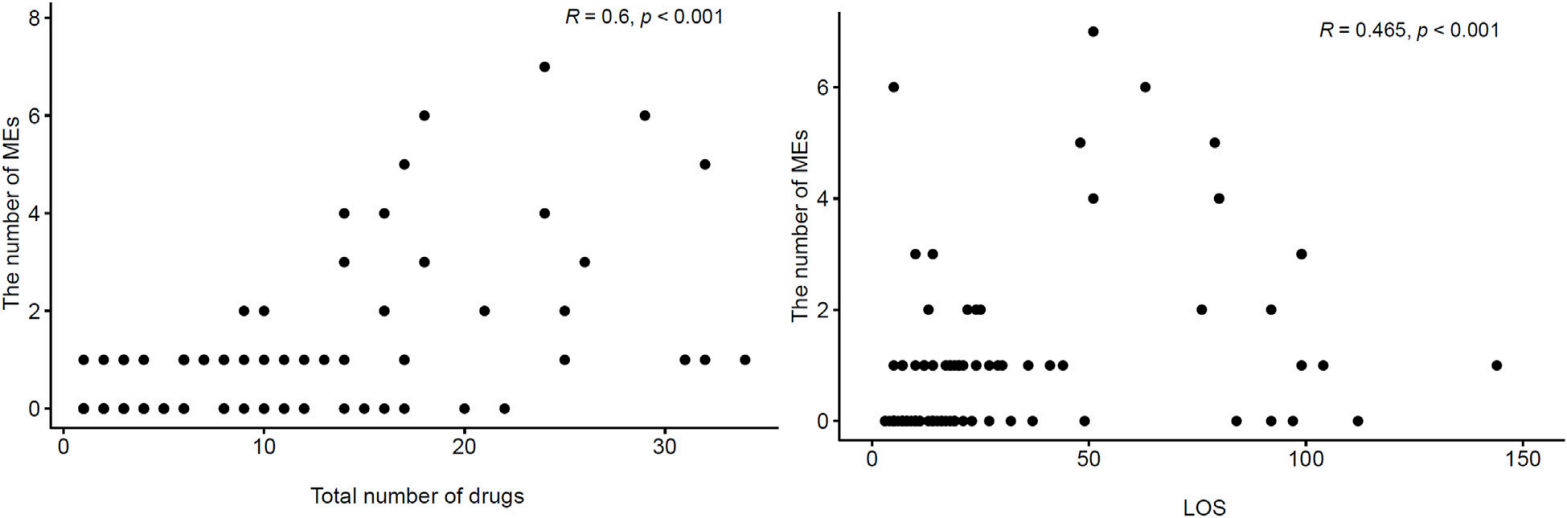
Scatter plots of total number of drugs, length of hospital stay and the number of medication errors.

**FIGURE 3:**
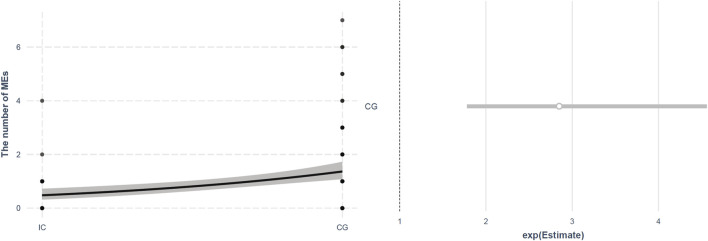
Plots of Poisson Regression Model for the number of medication errors.

When correlation analysis was performed for all patients, there was a moderate, positive correlation between the number of MEs and the total number of drugs (r = 0.600, *p*<0.001) and the LOS (r = 0.465, *p*<0.001) (Figure 2). When Poisson regression analysis was performed to compare both groups, the CG had 2.849 times more MEs than the IG (*p*<0.001) (Figure 3).

#### Adverse drug reactions

The number of patients with at least one ADR was 19 (36%) in the CG and 23 (47%) in the IG (*p*>0.05). However, there was no significant difference between both groups in terms of median number of ADRs (Table 4). Also, a total of 41 ADRs were detected in each group such as electrolyte imbalance, changes in blood count, nephrotoxicity, hepatotoxicity, disruption of the endocrine and cardiovascular systems (Supplementary Table S12).

**TABLE 4:** Comparison of adverse drug reactions (ADR) and drug-drug interactions (DDI) during hospitalization in both groups.

Prescription	Control group (n = 52)	Intervention group (n = 48)	*p* value
Presence of at least one ADR, n (%)	19 (36)	23 (47)	>0.05
Number of ADRs, median (range)	0 (0–6)	0 (0–5)	>0.05
The day of treatment that ADR was determined, median (range)	5 (1–28)	6 (1–60)	>0.05
Presence of at least one pDDI, n (%)	20 (38)	28 (58)	>0.05
Number of pDDIs, median (range)	0 (0–15)	1 (0–15)	0.012
Duration of exposure to pDDIs, median (range)	6.5 (1–28)	5 (1–28)	>0.05
Presence of at least one cDDI, n (%)	4 (7)	12 (25)	0.028
Number of cDDIs, median (range)	0 (0–4)	0 (0–5)	0.018
The day of treatment that cDDI was determined, median (range)	7 (1–28)	5 (1–28)	>0.05

pDDI, Potential drug-drug interactions; cDDI, Clinically significant drug-drug interactions.

When correlation analysis was performed for all patients, there was a strong, positive correlation between the number of ADRs and the total number of drugs (r = 0.672, *p*<0.001) and the LOS (r = 0.689, *p*<0.001).

#### Drug-drug interactions

In total, of the 185 pDDIs, 35 (18.9%) were classified as cDDI. The number of patients with at least one pDDI and cDDI were 20 (38%) and 4 (7%) in the CG, and 28 (58%) and 12 (25%) in the IG, respectively (Table 4). Although, there was no significant difference between both groups in terms of number of patients with at least one pDDI, there was significant difference in terms of number of patients with at least one cDDI, being more common in the IG (*p* = 0.028). Furthermore, more pDDIs and cDDIs were detected in the IG than in the CG during hospitalization (*p* = 0.012 and *p* = 0.018, respectively) (Table 4). Also, a total of 9 and 26 cDDIs were detected in the CG and IG respectively. These cDDI related to electrolyte imbalance, changes in plasma concentration, nephrotoxicity, disruption of the endocrine and cardiovascular systems (Supplementary Table S13).

When correlation analysis was performed for all patients, there was a stronger, positive correlation between the number of pDDIs and the total number of drugs (r = 0.849 vs. r = 0.562, *p*<0.001) and the LOS (r = 0.611 vs. r = 0.383, *p*<0.001) than the number of cDDIs. On the other hand, there was a strong, positive correlation between the duration of combination therapy and number of cDDIs (r = 0.656, *p*<0.001). When Poisson regression analysis was performed to compare both groups, the IG had 2.314 times higher probability of having a pDDIs and 3.734 times higher probability of having a cDDIs than the CG (*p*<0.001).

## Discussion

The benefits of clinical pharmacist intervention to lower DRPs or improving clinical outcomes were reported in nearly all observational clinical studies of neonatal pharmaceutical care (Simpson et al., 2004; Krzyzaniak and Bajorek, 2017). This study further adds to this information, but based on a randomized, controlled, double blind study evaluating the impact of clinical pharmacist-led services on determination, monitoring and intervention for DRPs (MEs, ADRs, and DDIs) in a NICU.

There were no significant differences in demographic and clinical characteristics between the two groups. However, although the rate of complications of labor and delivery and requiring multi-system support was higher in the CG, the higher rate of circulatory system diseases and surgery in the IG may have been the cause of the longer LOS in the IG.

Integrated in routine neonatal care, we observed that clinical pharmacist-provided practice notably enhanced prevention and management of MEs such as inappropriate drug selection, dose, preparation, and infusion time. On the other hand, while there was no difference between both groups in terms of incidence of ADRs detected by clinical pharmacist, pDDIs and cDDIs were found to be higher in the IG. Furthermore, we confirmed high DRPs incidence correlated with variety of prescribed drugs and LOS in line with current literature. During the study period, none of the patients in both groups had DRP that caused clinical outcomes such as serious harm, prolonged LOS or mortality.

In a ME incidence study published in 1987, neonatal patients, who were at that time less heterogenous and who were given a more limited number of drugs, experienced the lowest incidence of MEs (0.82/100 patient days) in two children’s hospitals (Folli et al., 1987). Since then, trends of medication use in the NICU evolved substantially over time especially in the last 10 years (Stark et al., 2022). The most common type of ME was inappropriate dosage (15%–82%) (Folli et al., 1987; Chedoe et al., 2007; Labib et al., 2018; Jafarian et al., 2019). In the current study, this was the most common physician-related ME (15% vs. 2%, CG to IG), but was considerably lower compared to previous studies (Folli et al., 1987; Chedoe et al., 2007; Labib et al., 2018; Jafarian et al., 2019). We assume that this is due to improvements in access to current and evidence-based formularies, clinical pharmacy services, and computerized physician order entry systems (Chedoe et al., 2007; Campino et al., 2008; Abbassi et al., 2022; Henry Basil et al., 2022; Shaniv et al., 2023). Reflecting on risk factors, Leopoldino et al. (2019) found that DRPs were associated with increased LOS and number of prescribed drugs (*p*<0.001).

There are some well-known studies reviewing the impact of clinical pharmacist-led education programmed in reducing ME (Simpson et al., 2004; Campino et al., 2008). However, appropriate pharmacotherapy requires daily clinical pharmacist-led systematically observation, prevention and subsequent intervention within a multidisciplinary NICU team. To reflect this setting, detailed data were obtained by examining each patient carefully at any time during the hospitalization process in the current RCT study.

Neonates cared for in the NICU are at higher risk of ADRs than other populations (Tice et al., 2020). Improvement of short- and long-term outcomes as well as reduction of health-related individual, family, and societal burdens can be significantly facilitated by early identification, quantification and mitigation of ADRs in the NICU (Samiee-Zafarghandy et al., 2023). Although there was no significant difference between the number and days of ADR determined by physicians-nurses and the clinical pharmacist in our study, the fact that 41 ADRs were only observed and determined by the clinical pharmacist clearly show the magnitude of the impact of clinical pharmacy services on ADR reports (Le et al., 2006).

According to the current literature, there are limited number of studies to identify and assess the neonatal DDIs compared to other populations (Costa et al., 2021; Rosen et al., 2021). Determination of more pDDIs and cDDIs with a clinical pharmacist-led monitoring compared to the CG enables or at least holds the promise to prevent and manage possible and severe ADRs. In our study, the main reason to follow the duration of exposure to a DDI as a cumulative effect was that it is associated with increased odds of ADRs such as acute kidney injury in physiological immaturity neonates with daily repeated monitoring (Salerno et al., 2021). At present, we found strong and positive correlation between the duration of exposure and number of cDDIs.

In this study, only clinical pharmacy services in the NICU were discussed. However, clinical pharmacy and clinical pharmacology have many reasons to work together to further patient-centered care related to pharmacotherapy (Burckart, 2012). Clinical pharmacologists are coordination of pharmacovigilance, TDM, pharmacoeconomics, provide consultation, conduct ethical and relevant clinical research (Cady, 1978). On the other hand, clinical pharmacists are delivering direct patient care and clinical practice, providing pharmaceutical services throughout medical center, and participating in pharmacy operations and medication dispensing. The collaboration of clinical pharmacy and clinical pharmacology keeps the science in patient-centered pharmacy services with their complementary skill sets, and that is essential for delivering the highest quality services as an optimal model. For this reason, we also strongly believe that collaboration between clinical pharmacists and clinical pharmacologists serves the integrity of different perspectives by providing rational pharmacotherapy in NICUs.

Because of the single-center study design, the small sample size, lack of detection of the rehospitalization rate, we are aware that this study has some limitations. The study period was not long enough to detect the long-term impact of a clinical pharmacist intervention on the impact of ADRs. Cost-effectiveness of the clinical pharmacist-led service was not explored. In addition, since ADRs and DDIs did not cause serious harm to patients, no intervention was made in order not to interrupt the efficacy, safety and tolerability of the pharmacotherapy. Larger, multi-center, cost-effective studies are required to assure the impact of the clinical pharmacist-led services in the personalized pharmaceutical care of NICU patients.

## Conclusion

The availability of a clinical pharmacist is effective to systematically prevent, identify and resolve DRPs (MEs, ADRs, DDIs) among NICU patients. The current study demonstrated that detailed clinical pharmacist observations and interventions in line with daily ward rounds enables the prevention and handling of DRPs.

## Data Availability

The data analyzed in this study is subject to the following licenses/restrictions: Data available on request due to privacy/ethical restrictions. Requests to access these datasets should be directed to nadir.yalcin@hacettepe.edu.tr.
