# Supplementary material for: Impact of clinical pharmacist-led intervention for drug-related problems in neonatal intensive care unit a randomized controlled trial
Source: Front Pharmacol. 2023 Aug 14;14:1242779. doi: 10.3389/fphar.2023.1242779 (PMC10461390; doi:10.3389/fphar.2023.1242779)
Supplement: Supplementary file 1 [file Table1.DOCX]

**Supplement 1.** Definition, examples and evaluation method of DRPs in each category

| **DRPs category** | **Definition** | **Examples** | **Evaluation method** | | |
| --- | --- | --- | --- | --- | --- |
|  |  |  | The neonatologist | The clinical pharmacist |  |
| Drug used without indication | Patient is taking a drug for no medically valid indication. | Although resistance to ampicillin developed, it was used for one more day. | √ | √√√ |  |
| Improper drug selection | Patient is not being treated with selected medication according to evidence-based medicine. | Although the culture result was negative, quadruple antimicrobial therapy (amikacin, vancomycin, meropenem, and fluconazole) was continued instead of meropenem monotherapy. | √ | √√√ |  |
| Subtherapeutic dose | Dosage causing no or minor change in the therapeutic responses. | Weight-based dosage was lower than the recommended by the current and evidence-based databases for ipratropium bromide. | √ | √√√ |  |
| Preparation | To prepare medicines taking into account patient/healthcare professional safety (contamination, hazardous agent, etc.) and drug stability | Gloves were not worn during receiving, unpacking, and placing in storage for spironolactone in line with NIOSH criteria. | √ | √√√ |  |
| Monitoring | It refers to the interpretation of physical, vital, and laboratory findings to maximize therapeutic benefit while minimizing toxicity. | Digoxin serum concentration was not obtained within 5 to 7 days (approximate time to steady-state). | √ | √√√ |  |
| Adverse drug reactions | Patient has a medical problem that is the result of adverse drug reactions. | - Morphine related respiratory depression was determined.  - Isotretinoin related anemia and neutropenia was determined. | √ | √ |  |
| Drug-drug interactions | Patient has a medical problem that is the result of potential drug-drug interaction. | - Phenobarbital + levetiracetam related decrease the effect of levetiracetam was determined.  - Captopril + enoxaparin related hyperkalemia was determined. | √ | √ |  |

NIOSH: National Institute for Occupational Safety & Health

√: only monitoring as observer, √√√: monitoring, prevention and intervention

**Supplement 2.** Operational Definitions of Infant Acuity Levels Originally Described by AAP/ACOG

| **1** | *Continuing care* | Infant only requiring PO or NG feedings, occasional enteral medications, basic monitoring. May or may not have a heparin lock for meds. |
| --- | --- | --- |
| **2** | *Requiring intermediate care* | Stable infant on established management plan, not requiring significant support. Examples would include: Room air, supplemental oxygen or low flow nasal cannula, several meds. |
| **3** | *Requiring intensive care* | Infant is stabilized, though requires frequent treatment and monitoring to assure maintenance of stability. Examples would include: Ventilator, CPAP, high-flow nasal cannula, multiple IV meds via central or peripheral line. |
| **4** | *Requiring multi-system support* | Infant requires continuous monitoring and interventions. Examples would include: Conventional ventilation, stable on HFV, continuous drug infusions, several IV fluid changes via central line. |
| **5** | *Unstable, requiring complex critical care* | Infant is medically unstable and vulnerable, requiring many simultaneous interventions. Examples would include: ECMO, HFV, nitric oxide, frequent administration of fluids, medication. |

AAP/ACOG: *American Academy of Pediatrics* & *American College of Obstetricians and Gynecologists* (1992; 2007), PO: by mouth, NG: nasogastric, CPAP: continuous positive airway pressure, IV: intravenous, HFV: high-frequency ventilation, ECMO: extracorporeal membrane oxygenation

**Supplement 3.** Medication Error Checklist for NICU Patients

|  | **Day 1** | **Day 2** | **Day 3** | **Day 4** | **Day 5** | **Day 6** | **Day 7** | **Day 8** | **Day 9** | **Day 10** | **Day 11** | **Day 12** | **Day 13** | **Day 14** |
| --- | --- | --- | --- | --- | --- | --- | --- | --- | --- | --- | --- | --- | --- | --- |
| **Prescription** |  | | | | | | | | | | | | | |
| *Inappropriate drug* |  |  |  |  |  |  |  |  |  |  |  |  |  |  |
| *Inappropriate unit* |  |  |  |  |  |  |  |  |  |  |  |  |  |  |
| *Inappropriate dose* |  |  |  |  |  |  |  |  |  |  |  |  |  |  |
| *Inappropriate dose interval* |  |  |  |  |  |  |  |  |  |  |  |  |  |  |
| *Inappropriate infusion rate* |  |  |  |  |  |  |  |  |  |  |  |  |  |  |
| **Preparation** |  | | | | | | | | | | | | | |
| *Inappropriate drug* |  |  |  |  |  |  |  |  |  |  |  |  |  |  |
| *Inappropriate occupational safety* |  |  |  |  |  |  |  |  |  |  |  |  |  |  |
| *Inappropriate storage* |  |  |  |  |  |  |  |  |  |  |  |  |  |  |
| **Administration** |  | | | | | | | | | | | | | |
| *Dose omission* |  |  |  |  |  |  |  |  |  |  |  |  |  |  |
| *Extra dose* |  |  |  |  |  |  |  |  |  |  |  |  |  |  |
| *Inappropriate time* |  |  |  |  |  |  |  |  |  |  |  |  |  |  |
| *Inappropriate infusion* |  |  |  |  |  |  |  |  |  |  |  |  |  |  |
| *Inappropriate technique* |  |  |  |  |  |  |  |  |  |  |  |  |  |  |
| **Monitoring** |  | | | | | | | | | | | | | |
| *Physical* |  |  |  |  |  |  |  |  |  |  |  |  |  |  |
| *Vital* |  |  |  |  |  |  |  |  |  |  |  |  |  |  |
| *Laboratory* |  |  |  |  |  |  |  |  |  |  |  |  |  |  |
| *TDM* |  |  |  |  |  |  |  |  |  |  |  |  |  |  |

* The checklist can be expanded according to the duration of the patient's hospitalization.

** The day ME is detected should be ticked

TDM: Therapeutic drug monitoring

**Supplement 4.** New Adverse Drug Reactions Algorithm for Infants in Neonatal Intensive Care Units (The Du’s Tool) *

| **ADRs Assessment Criteria** | **Yes** | **No** | **N/A** |
| --- | --- | --- | --- |
| 1. Was the timing of AE consistent with an ADR to the suspected drug? | 6 | -7 | 0 |
| 1. Is the AE a well-documented ADR to the suspected drug? | 0 | -6 | 0 |
| 1. Are there published reports on this AE that are related to the suspected drug in newborns? | 4 | -4 | 0 |
| 1. Was the AE likely a change (exacerbation, recurrence, complication, or new manifestation) in a preexisting clinical condition? | -3 | 7 | 0 |
| 1. Are there any alternative etiologic candidates other than the preexisting condition (e.g., concomitant drugs) that are a common cause of the AE? | -3 | 2 | 0 |
| 1. Was an alternative etiologic candidate confirmed by any objective evidence? | -3 | 3 | 0 |
| 1. Did the AE improve after suspected drug was discontinued? | 4 | -1 | 0 |
| 1. Was the AE less severe when the dose was reduced? | 4 | -2 | 0 |
| 1. Did the AE improve after a specific antagonist was administered? | 3 | -1 | 0 |
| 1. Did the AE significantly diminish or disappear while patient was still taking the suspected drug? | -2 | 1 | 0 |
| 1. Did the AE reappear/worsen when suspected drug was reintroduced? | 9 | -1 | 0 |
| 1. Was the suspected drug detected in blood or other fluids in concentrations known to be toxic? | 4 | -2 | 0 |
| 1. Is there unequivocal evidence that the amount of the suspected drug received was an overdose for this patient? | 4 | -4 | 0 |

* Du, W., Lehr, V.T., Lieh-Lai, M., Koo, W., Ward, R.M., Rieder, M.J., et al. (2013). An algorithm to detect adverse drug reactions in the neonatal intensive care unit*. J Clin Pharmacol* 53(1), 87-95. doi: 10.1177/0091270011433327.

ADR: Adverse drug reaction, AE: Adverse event, N/A: Not applicable

Total Score = _________

Category = ___________

If Total Score ≥ 14 **→**Definite

If 7 ≤ Total Score ≤ 13 **→**Probable

If 3 ≤ Total Score ≤ 6 **→**Possible

If Total Score ≤ 2 **→**Unlikely

**Supplement 5.** Generic severity criteria of International Neonatal Consortium Neonatal Adverse Event Severity scale developed for use in neonates *

| **Grade 1** | **Grade 2** | **Grade 3** | **Grade 4** | **Grade 5** |
| --- | --- | --- | --- | --- |
| Mild | Moderate | Severe | Life threatening | Death |
| Mild; asymptomatic or mild symptoms; clinical or diagnostic observations only; intervention not indicated. | Moderate; minimal, local or non- invasive intervention indicated; limiting age-appropriate instrumental activities of daily living. | Severe or medically significant but not immediately life threatening; hospitalization or prolongation of hospitalization indicated; disabling; limiting self-care activities of daily living. | Life-threatening consequences; urgent intervention indicated. | Death related to adverse event. |

* Salaets, T., Turner, M.A., Short, M., Ward, R.M., Hokuto, I., Ariagno, R.L., et al. (2019). Development of a neonatal adverse event severity scale through a Delphi consensus approach. Arch Dis Child 104(12), 1167-1173. doi: 10.1136/archdischild-2019-317399.

| **Date** | **Drug** | **Dose** | **Administration route** | **The day of treatment** | **ADR** | **Du’s Category** | **Severity** |
| --- | --- | --- | --- | --- | --- | --- | --- |
|  |  |  |  |  |  |  |  |
|  |  |  |  |  |  |  |  |
|  |  |  |  |  |  |  |  |
|  |  |  |  |  |  |  |  |
|  |  |  |  |  |  |  |  |
|  |  |  |  |  |  |  |  |
|  |  |  |  |  |  |  |  |
|  |  |  |  |  |  |  |  |
|  |  |  |  |  |  |  |  |

**Supplement 6.** Adverse Drug Reactions Follow-up Form

**Supplement 7.** Drug Interaction Probability Scale (DIPS) *

| **Questions** | **Yes** | **No** | **Unknown or N/A** |
| --- | --- | --- | --- |
| 1. Are there previous credible reports of this interaction in humans? | +1 | -1 | 0 |
| 1. Is the observed interaction consistent with the known interactive properties of precipitant drug? | +1 | -1 | 0 |
| 1. Is the observed interaction consistent with the known interactive properties of object drug? | +1 | -1 | 0 |
| 1. Is the event consistent with the known or reasonable time course of the interaction (onset and/or offset)? | +1 | -1 | 0 |
| 1. Did the Interaction remit upon dechallenge of the precipitant drug with no change in the object drug? (If no dechallenge, use Unknown or N/A and skip Question 6) | +1 | -2 | 0 |
| 1. Did the Interaction reappear when the precipitant drug was readministered in the presence of continued use of object drug? | +2 | -1 | 0 |
| 1. Are there reasonable alternative causes for the event? | -1 | +1 | 0 |
| 1. Was the object drug detected in the blood or other fluids in concentrations consistent with the proposed interaction? | +1 | 0 | 0 |
| 1. Was the drug interaction confirmed by any objective evidence consistent with the effects on the object drug (other than drug concentrations from question 8)? | +1 | 0 | 0 |
| 1. Was the interaction greater when the precipitant drug dose was increased or less when the precipitant drug dose was decreased? | +1 | -1 | 0 |

* Horn, J.R., Hansten, P.D., and Chan, L.N. (2007). Proposal for a new tool to evaluate drug interaction cases. *Ann Pharmacother* 41(4), 674-680. doi: 10.1345/aph.1H423.

Total Score = _________

Category = ___________

If Total Score > 8 **→**Highly Probable

If 5 ≤ Total Score < 8 **→**Probable

If 2 ≤ Total Score ≤ 4 **→**Possible

If Total Score < 2 **→**Doubtful

**Supplement 8.** *UpToDate (Lexicomp^®^)* Drug Interaction Severity

| **Risk rating** | **Description** |
| --- | --- |
| X (Avoid combination) | Data demonstrate that the specified agents may interact with each other in a clinically significant manner. The risks associated with concomitant use of these agents usually outweigh the benefits. These agents are generally considered contraindicated. |
| D (Consider therapy modification) | Data demonstrate that the two medications may interact with each other in a clinically significant manner. A patient-specific assessment must be conducted to determine whether the benefits of concomitant therapy outweigh the risks. Specific actions must be taken in order to realize the benefits and/or minimize the toxicity resulting from concomitant use of the agents. These actions may include aggressive monitoring, empiric dosage changes, choosing alternative agents. |
| C (Monitor therapy) | Data demonstrate that the specified agents may interact with each other in a clinically significant manner. The benefits of concomitant use of these two medications usually outweigh the risks. An appropriate monitoring plan should be implemented to identify potential negative effects. Dosage adjustments of one or both agents may be needed in a minority of patients. |
| B (No action needed) | Data demonstrate that the specified agents may interact with each other, but there is little to no evidence of clinical concern resulting from their concomitant use. |
| A (No known interaction) | Data have not demonstrated either pharmacodynamic or pharmacokinetic interactions between the specified agents |

| **Date** | **Potential DDI** | **Inhibitor/Inductor** | **Expected** | **Actualized** | **The day of treatment** | **Duration of exposure** | **DIPP Category** | **Severity** |
| --- | --- | --- | --- | --- | --- | --- | --- | --- |
|  |  |  |  |  |  |  |  |  |
|  |  |  |  |  |  |  |  |  |
|  |  |  |  |  |  |  |  |  |
|  |  |  |  |  |  |  |  |  |
|  |  |  |  |  |  |  |  |  |
|  |  |  |  |  |  |  |  |  |
|  |  |  |  |  |  |  |  |  |
|  |  |  |  |  |  |  |  |  |
|  |  |  |  |  |  |  |  |  |

**Supplement 9.** Potential and Clinically Significant Drug-Drug Interactions Follow-up Form

**Supplement 10.** Pharmacological groups for prescribed each medication according to the Anatomical Therapeutic Chemical classification

| **Anti-infectives for systemic use** | Amikacin  Amoxicillin  Ampicillin  Ampicillin-sulbactam  Azithromycin  Cefazolin  Cefotaxime  Ceftriaxone  Cefuroxime  Ciprofloxacin  Colistin  Fluconazole | Gentamicin  Imipenem  Linezolid  Meropenem  Metronidazole  Ornidazole  Oseltamivir  Penicillin G  Piperacillin-tazobactam  Teicoplanin  Vancomycin |
| --- | --- | --- |
| **Systemic hormonal preparations** | Desmopressin  Dexamethasone  Glucagon  Hydrocortisone  Insulin  Levothyroxine | Methylprednisolone  Octreotide  Pancreatic  Prednisolone  Terlipressin |
| **Nervous system** | Acetaminophen  Caffeine  Dexmedetomidine  Diazepam  Fentanyl  Ketamine | Levetiracetam  Midazolam  Morphine  Phenobarbital  Phenytoin |
| **Blood and blood-forming organs** | Albumin  Acetylsalicylic acid  Enoxaparin  Ferrous fumarate  Fibrinogen | Heparin  Immunoglobulin  Tranexamic acid  Vitamin K |
| **Alimentary tract and metabolism** | Aluminum hydroxide  Calcitriol  Calcium salts  Carnitine  H_2_-blockors  IV solutions  Magnesium salts  Multivitamins | Potassium salts  Probiotics  Proton pump inhibitors  Shohl’s solutions  Sodium salts  Ursodiol  Vitamin A, B, C, D  Zinc supplements |
| **Cardiovascular system** | Adenosine  Alprostadil  Amiodarone  Amlodipine  Captopril  Diazoxide  Dopamine  Epinephrine  Flecainide  Furosemide  Hydrochlorothiazide | Ibuprofen  Levosimendan  Methylene blue  Milrinone  Nifedipine  Nitroglycerin  Norepinephrine  Propranolol  Sotalol  Spironolactone |
| **Respiratory system** | Acetylcysteine  Dornase alpha  Ipratropium | Salbutamol (albuterol)  Surfactant |
| **Sensory organs** | Dexamethasone eyedrop  Enema  Latanoprost eyedrop  Mupirocin | Netilmicin eyedrop  Nitrofurazone  Ophthalmic lubricant  Tobramycin |

**Supplement 11.** Medication errors that were reported to and accepted by the clinical pharmacist for the intervention group patients (sum=40)

| **Type of MEs** | **By whom** | **Category*** | **Problems **** | **Intervention** |
| --- | --- | --- | --- | --- |
| Logistic problems | Physicians | B | Amikacin was not available in the pharmaceutical market (2). | Recommended to use gentamicin as an alternative |
| Drug preparation | Nurses | B | It was determined that the vitamin A capsule was prepared incorrectly. | Recommended to correct method of preparation in accordance with guidelines. |
| Duration of infusion for loading dose | Nurses | C | Fluconazole infusion time was determined to be less than one hour (8). | Recommended to be infused at concentration of 2 mg/mL over 1 to 2 hours. |
| Drug used without indication | Physicians | C | Developed resistance to ampicillin. | Recommended to stop the ampicillin. |
| Administration technique | Nurses | C | Propranolol immediate-release was not administered on an empty stomach. | Recommended to take it half an hour before feeding. |
| Improper medication use | Physicians | D | It was determined that alprostadil was administered for more than 120 hours (risk of antral hyperplasia and gastric outlet obstruction). | Recommended to stop the alprostadil. |
| High cost | Physicians | B | High-cost (₺1703.25) propranolol solution was considered to be administered in a patient with infantile hemangiomas. | Recommended to crush the lower cost propranolol tablet (₺36.08) instead. |
| Administration time | Nurses | C | Prednisolone prescribed once daily was administered in the evening. | Recommended to administered in the morning (it mimics the timing of their body's own production of cortisone in the morning). |
| Duration of infusion | Nurses | C | Intravenous immunoglobulin administered by rapid infusion (<2 hours). | Recommended to infuse as slowly as indication and stability allow over 2 hours. |
| Improper drug selection | Physicians | D | Although the culture result was negative, quadruple antimicrobial therapy (amikacin, vancomycin, meropenem, and fluconazole) was continued. | Recommended to continue monotherapy with meropenem. |
| **Supplement 11 *cont.*** | | | | |
| Dosing in medication order | Physicians | B | The dose for dexmedetomidine was misspelled. | Recommended to prescribe the correct dose. |
| Monitoring | Physicians | C | Antifactor Xa was not monitored in a patient who had been administered enoxaparin for a long time. | Recommended to monitoring antifactor Xa. |
| Dosing in medication order | Physicians | C | Vancomycin and amikacin plasma (trough) levels were found to be low. | Recommended to dose adjustment in accordance with the postnatal age and weight of the patient. |
| Dosing in medication order and dose interval | Physicians | C | Dosing was not individualized to patient response to achieve and maintain target serum glucose concentrations. Also, dose interval was divided every 12 hours instead of 6 to 8 hours. | Recommended to increasing the dose and reducing the dosing interval to every 6 hours. |
| Dosing in medication order and dose interval | Physicians | C | Weight-based dosage and the dose interval were inappropriate for ipratropium bromide. | Recommended to increasing the dose and reducing the dosing interval to every 8 hours. |
| Storage | Nurses | B | The prepared diazoxide suspension was not shaken before administration. | Recommended to shake to avoid possible clogging in the orogastric tube. |
| Duration of infusion | Nurses | C | Alteplase administered short-term into the lumen (2). | Recommended to alteplase clamp drain for 1 to 2 hours in the occluded IV catheter |
| Administration technique | Nurses | C | Contents removed from isotretinoin softgel capsule may irritate esophagus in a patient with neuroblastoma. | Recommended to puncture the capsule with a needle, then mix the contents of the capsule with 5 to 10 mL of milk or formula and draw the mixture up into oral syringe and administer via feeding tube. |
| Administration technique | Nurses | C | Sildenafil oral suspension was not shaken prior to use and was not protected at 2°C to 8°C. | Recommended to shake prior to use and store in the refrigerator. |
| Administration technique | Nurses | C | Enalapril and captopril immediate-release were not administered on an empty stomach (3). | Recommended to take it half an hour before feeding. |
| **Supplement 11 *cont.*** | | | | |
| Administration technique | Nurses | C | Pantoprazole granules were administered directly to the nasogastric tube. | Recommended to empty the granules into the syringe barrel, add 5 mL of sterile water, and tap/shake the barrel of the syringe to aid in emptying the syringe. |
| Monitoring | Physicians | C | Digoxin serum concentration did not obtain within 5 to 7 days (approximate time to steady-state). | Recommended to monitoring serum concentration of digoxin. |
| Monitoring | Physicians | C | Drug-related problems with flecainide were detected. | Recommended to adjusted of the dose interval of flecainide, administration on an empty stomach and therapeutic drug monitoring. |
| Preparation | Nurses | C | Enteric-coated aspirin was administered by crushing (2). | Recommended to use uncoated aspirin. |
| Monitoring | Physicians | B | No plasma level follow-up was performed in the patient who had been on phenobarbital for a long time (2). | Recommended to monitoring serum concentration of phenobarbital. |
| Monitoring | Physicians | B | Peak serum concentration of amikacin was observed instead of trough levels. | Recommended to obtain trough concentration at the end of a dosing interval. |
| Preparation | Nurses | B | Gloves were not worn during receiving, unpacking, and placing in storage for spironolactone. | Recommended to worn gloves (single) in line with NIOSH recommendations. |

NIOSH: The National Institute for Occupational Safety and Health

* US NCC MERP (National Coordinating Council for Medication Error Reporting and Prevention) Index for Categorizing Medication Errors: B=No harm, an error occurred but the error did not reach the patient; C=No harm, an error occurred that reached the patient but did not cause patient harm, D=No harm, an error occurred that reached the patient and required monitoring to confirm that it resulted in no harm to the patient and/or required intervention to preclude harm.

** The numbers in parentheses indicate how many patients in the intervention group had the mentioned DRPs.

**Supplement 12.** Comparison of reported adverse drug reactions in both groups

| **Control group (sum=41)** | **Intervention group (sum=41)** |
| --- | --- |
| Spironolactone-hydrochlorothiazide related hypercalcemia and hyponatremia on the 7^th^ day of treatment (G1). | Amiodarone related hemolysis on the 30^th^ day of treatment (G3). |
| Furosemide related hypochloremia on the 4^th^-19^th^ day of treatment (G2) (4). | Flecainide related AV block and wide QRS on the 26^th^ day of treatment (G4). |
| Desmopressin related hyponatremia on the 1^st^ day of treatment (G1). | Salbutamol related hypokalemia on the 12^th^ day of treatment (G1). |
| Metronidazole related AST elevation on the 1^st^-5^th^ day of treatment (G2) (3). | Enalapril related hyperkalemia on the 8^th^ day of treatment (G1). |
| Dexamethasone related hyperglycemia on the 3^rd^-8^th^ day of treatment (G1) (4). | Amiodarone related TSH elevation on the 60^th^ day of treatment (G2). |
| Dexamethasone related hypertension on the 5^th^ day of treatment (G2). | Phenobarbital related anemia on the 2^nd^ day of treatment (G2). |
| Morphine related respiratory depression on the 2^nd^ day of treatment (G4). | Carbamazepine related GGT elevation on the 1^st^ day of treatment (G2). |
| Milrinone related hypotension on the 2^nd^ day of treatment (G3). | Captopril related hyperkalemia on the 21^st^ day of treatment (G1) (2). |
| Dopamine related hypertension on the 3^rd^ day of treatment (G2). | Enoxaparin related hematuria on the 7^th^ day of treatment (G4). |
| Phenytoin and phenobarbital related hepatotoxicity on the 14^th^ day of treatment (G3). | Furosemide related hypokalemia on the 2^nd^ day of treatment (G1) (3). |
| Hydrocortisone related hyperglycemia on the 3^rd^ day of treatment (G1). | Dexamethasone related hyperglycemia on the 3^rd^ day of treatment. |
| Parenteral nutrition related hyperglycemia on the 6^th^-13^th^ day of treatment (G1) (3). | Furosemide related hypochloremia on the 7^th^-20^th^ day of treatment (G2) (3). |
| Spironolactone related hyperkalemia on the 5^th^ day of treatment (G2). | Spironolactone related hyperkalemia on the 28^th^ day of treatment (G1). |
| Hydrochlorothiazide related hypochloremia on the 5^th^ day of treatment (G1). | Enalapril related hyperkalemia on the 18^th^ day of treatment (G1). |
| Propranolol related reflex tachycardia on the 3^rd^ day of treatment (G3). | Furosemide related hypocalcemia on the 3^rd^-5^th^ day of treatment (G1) (3). |
| **Supplement 12 *cont*.** | |
| Propranolol related bradycardia and hypotension on the 12^th^ day of treatment (G2). | Parenteral nutrition related hyperglycemia on the 2^nd^ day of treatment (G1). |
| Ibuprofen related thrombocytopenia on the 6^th^ day of treatment (G2). | Enoxaparin related INR elevation on the 6^th^ day of treatment (G3). |
| Furosemide related hyponatremia on the 5^th^ day of treatment (G1). | Diazoxide related hyperglycemia on the 10^th^ day of treatment (G2). |
| Levothyroxine related TSH over-suppression on the 20^th^ day of treatment (G2). | Linezolid related anemia, thrombocytopenia, lactate elevation on the 6^th^-12^th^ day of treatment (G3). |
| Vancomycin related neutropenia on the 27^th^ day of treatment (G3). | Vancomycin related neutropenia on the 5^th^-21^st^ day of treatment (G3) (2). |
| Meropenem related eosinophilia on the 27^th^ day of treatment (G2). | Parenteral nutrition related cholestasis on the 60^th^ day of treatment (G3). |
| Fentanyl related hypotension on the 2^nd^ day of treatment (G2). | Fluconazole related AST elevation and hypokalemia on the 1^st^ day of treatment (G2) (3). |
| Dobutamine related hypertension on the 4^th^ day of treatment (G1). | Zidovudine related neutropenia on the 9^th^ day of treatment (G3). |
| Parenteral nutrition related cholestasis on the 21^st^-24^th^ day of treatment (G3) (2). | Adrenaline related sinus tachycardia on the 2^nd^ day of treatment (G3). |
| Enoxaparin related hematuria on the 4^th^ day of treatment (G3). | Caffeine related hyponatremia on the 1^st^-12^th^ day of treatment (G1) (3). |
| Parenteral nutrition related hyperkalemia on the 3^rd^ day of treatment (G1) (3). | Alprostadil related hypotension on the 1^st^ day of treatment (G2). |
| Caffeine related hyponatremia on the 1^st^-14^th^ day of treatment (G1) (2). | Dexmedetomidine related hypotension on the 1^st^ day of treatment (G2). |
|  | Midazolam related myoclonus on the 1^st^ day of treatment (G4). |
|  | Isotretinoin related anemia and neutrophilia on the 8^th^ day of treatment (G3). |

AV: Atrioventricular, AST: Aspartate aminotransferase, GGT: Gamma-glutamyl transferase,

TSH: thyroid stimulating hormone, INR: International normalized ratio

* The numbers in parentheses indicate how many patients in the both groups had the ADRs.

According to the Du’s tool category (causality scale), all ADRs were in the "definite" category.

According to the neonatal adverse event severity scale, severity was subdivided into five grades (G): mild (G1), moderate (G2), severe (G3), life threatening (G4) and death (G5).

**Supplement 13.** Comparison of reported clinically significant drug-drug interactions in both groups

| **Control group (sum=9)** | **Intervention group (sum=26)** |
| --- | --- |
| Methylprednisolone + fluconazole related hypertension on the 1^st^ day of combination (C). | Dexmedetomidine + fentanyl related hypotension on the 3^rd^ day of combination (C) (2). |
| Phenobarbital + levetiracetam related decrease the effect of levetiracetam on the 7^th^ day of combination (C). | Amikacin + vancomycin related creatinine elevation on the 7^th^-28^th^ day of combination (C) (3). |
| Dexmedetomidine + phenobarbital related hypotension on the 3^rd^ day of combination (C). | Adenosine + dexmedetomidine related bradycardia on the 2^nd^ day of combination (C). |
| Furosemide + phenobarbital related hypotension on the 3^rd^ day of combination (C). | Amikacin + furosemide related creatinine elevation on the 5^th^ day of combination (C). |
| Dexmedetomidine + sildenafil related hypotension on the 2^nd^ day of combination (C). | Phenobarbital + levetiracetam related decrease the effect of levetiracetam on the 20^th^ day of combination (C). |
| Levothyroxine + ciprofloxacin related T4 decrease on the 10^th^ day of combination (C). | Dexmedetomidine + furosemide related hypotension on the 1^st^-3^rd^ day of combination (C) (3). |
| Topiramate + phenytoin related increase the serum concentration of phenytoin on the 1^st^ day of combination (C). | Captopril + spironolactone related hypokalemia on the 20^th^ day of combination (C). |
| Levothyroxine + phenobarbital related T4 decrease on the 12^th^ day of combination (C). | Phenobarbital + phenytoin related increase the serum concentration of phenobarbital on the 7^th^ day of combination (C). |
| Phenobarbital + phenytoin related decrease the serum concentration of phenytoin on the 12^th^ day of combination (C). | Phenobarbital + phenytoin related decrease the serum concentration of phenytoin on the 16^th^ day of combination (C). |
|  | Phenobarbital + levetiracetam related increase the serum concentration of phenobarbital on the 20^th^ day of combination (C). |
|  | Dexmedetomidine + levosimendan related hypotension on the 2^nd^ day of combination (C). |
|  | Spironolactone + potassium chloride related hyperkalemia on the 3^rd^ day of combination (D). |
| **Supplement 13 *cont*.** | |
|  | Furosemide + levosimendan related hypotension on the 2^nd^ day of combination (C). |
|  | Dexmedetomidine + fentanyl related bradycardia on the 2^nd^ day of combination (C). |
|  | Captopril + enoxaparin related hyperkalemia on the 2^nd^ day of treatment (C). |
|  | Phenytoin + fluconazole related increase the serum concentration of phenytoin on the 5^th^ day of combination (C). |
|  | Enalapril + enoxaparin related hyperkalemia on the 7^th^ day of treatment (C). |
|  | Captopril + furosemide related hypotension on the 5^th^ day of combination (C). |
|  | Dexmedetomidine + digoxin related bradycardia on the 3^rd^ day of combination (C). |
|  | Furosemide + hydrocortisone related hypokalemia on the 3^rd^ day of combination (C). |
|  | Digoxin + sodium bicarbonate related decrease the serum concentration of digoxin on the 5^th^ day of combination (B). |

Risk Ratings (Lexicomp Drug Interactions); X: Avoid combination, D: Consider therapy modification, C: Monitor therapy,

B: No action needed, and A: No known interaction.

* The numbers in parentheses indicate how many patients in the both groups had the cDDIs.

According to the drug interaction probability scale (severity), all cDDIs were in the "highly probable" category.
